# Can risk modelling improve treatment decisions in asymptomatic carotid stenosis?

**DOI:** 10.1186/s12883-019-1528-7

**Published:** 2019-11-22

**Authors:** James F. Burke, Lewis B. Morgenstern, Rodney A. Hayward

**Affiliations:** 1 Deparment of Neurology, University of Michigan, Ann Arbor, USA; 2 Deparment of Neurology, Ann Arbor VA, Ann Arbor, USA; 3 Deparment of Internal Medicine, University of Michigan, Ann Arbor, USA; 4 The Center for Clinical Management and Research, Ann Arbor VA, Ann Arbor, USA; 5 RWJ Clinical Scholars Program, Room G100-36, Building 14, NCRC, 2800 Plymouth Rd, Ann Arbor, MI 48109 USA

**Keywords:** Carotid endarterectomy, Asymptomatic carotid stenosis, Risk prediction

## Abstract

**Background:**

Carotid endarterectomy (CEA) is routinely performed for asymptomatic carotid stenosis, yet its average net benefit is small. Risk stratification may identify high risk patients that would clearly benefit from treatment.

**Methods:**

Retrospective cohort study using data from the Asymptomatic Carotid Atherosclerosis Study (ACAS). Risk factors for poor outcomes were included in backward and forward selection procedures to develop *baseline risk models* estimating the risk of non-perioperative ipsilateral stroke/TIA. Baseline risk was estimated for all ACAS participants and externally validated using data from the Atherosclerosis Risk in Communities (ARIC) study. Baseline risk was then included in a *treatment risk model* that explored the interaction of baseline risk and treatment status (CEA vs. medical management) on the patient-centered outcome of any stroke or death, including peri-operative events.

**Results:**

Three baseline risk factors (BMI, creatinine and degree of contralateral stenosis) were selected into our baseline risk model (c-statistic 0.59 [95% CI 0.54–0.65]). The model stratified absolute risk between the lowest and highest risk quintiles (5.1% vs. 12.5%). External validation in ARIC found similar predictiveness (c-statistic 0.58 [0.49–0.67]), but poor calibration across the risk spectrum. In the treatment risk model, CEA was superior to medical management across the spectrum of baseline risk and the magnitude of the treatment effect varied widely between the lowest and highest absolute risk quintiles (3.2% vs. 10.7%).

**Conclusion:**

Even modestly predictive risk stratification tools have the potential to meaningfully influence clinical decision making in asymptomatic carotid disease. However, our ACAS model requires target population recalibration prior to clinical application.

## Background

Asymptomatic carotid endarterectomy (CEA) is commonly performed, yet, considerable uncertainty exists about the magnitude of benefit and which patients should be treated [1]. The best estimates of the risk-benefit ratio of CEA are derived from trials performed in the 1990s [2, 3]. These trials found a consistent, but small, average absolute reduction in stroke risk, approximately 1 in 100 people per year.

For a variety of conditions and treatments, the risks and benefits of treatment can vary markedly between higher and lower risk patients [4, 5]. For example, in symptomatic carotid stenosis, patients with the highest stroke risk have more than a 50% risk of stroke over 2 years whereas those with the lowest risk have less than a 10% risk [6]. Assuming, as seems to be the case, that CEA has similar relative benefits across risk groups, the highest risk patients would have more than 30 in 100 risk reduction over 2 years (1 stroke prevented after 2 years per no more than 3 CEAs [NNT < 3]) vs. a much smaller net benefit in the lowest risk patients (NNT > 15) [7]. Given the relatively high stroke risk of symptomatic patients, however, even relatively lower risk patients may receive a meaningful net benefit from CEA, but this may not be true for lower risk asymptomatic patients. If, as with symptomatic carotid stenosis, higher risk patients have a larger benefit from treatment than lower risk patients, then a paradigm of CEA for higher-risk asymptomatic patients and medical management for lower-risk patients could address much of the asymptomatic carotid treatment controversy. For this reason, tools to stratify asymptomatic carotid risk have been established as a high research priority [8].

To inform tools to stratify asymptomatic CEA risk, we sought to determine how baseline risk of important outcomes varies in the Asymptomatic Carotid Atherosclerosis Study (ACAS).

### Hypothesis

We hypothesized that: 1. clinically meaningful variation in risk would exist in ACAS and readily be predicted by baseline clinical factors and 2. No variation in relative treatment benefit would exist across the risk spectrum, but considerable variation in absolute benefit would exist.

## Methods

### Overview and rationale

ACAS was a clinical trial that randomized patients to CEA or medical management in a 1:1 ratio in the context of 60% or greater asymptomatic carotid stenosis [2]. ACAS data is available, after application, from the National Institute of Neurological Disorders and Stroke (NINDS) archived clinical research datasets repository [9]. We used ACAS data to explore how clinical factors, measured at study entry, predict outcomes in medically and surgically treated patients by first incorporating these factors into a *baseline risk model*. After stratifying patients by baseline risk, we then explored how patient-centered outcomes varied across baseline risk using a *treatment outcomes model.*

A limitation of using ACAS to study carotid risk stratification is that both medical and surgical management have evolved considerably over the 20 years since ACAS’s publication. Yet, ACAS is a reasonable dataset to explore our primary hypotheses for two reasons. First, without randomization, it is impossible to determine whether medical and surgical treatment work differently in high and low risk patients. While observational studies provide insights into risk factors, non-random treatment assignment limits any inferences about the relationship between risk and treatment status. Second, while it is likely that absolute stroke and death rates have decreased since ACAS, for both medically and surgically treated patients, it is less likely that the relative relationship between risk factors and event rates has evolved. For example, because the absolute mortality rates in statin trials range from 0 to 20% based on the trial timing and population, there is wide variation in the absolute risk reductions from 0% per year to over 2% per year even though the relative effect sizes of statins are fairly consistent across studies [10]. If similar logic applies to CEA then, then while the absolute event rates in ACAS would likely overestimate the event rates in modern care, a patient that was “high risk” in ACAS would still be “high risk” today, even though the absolute level of risk would likely be lower today.

To test our first hypothesis, that risk for adverse outcomes would vary over the ACAS population, we built a *baseline risk model* to determine how effectively multi-variable risk factor models would predict the risk of ipsilateral stroke/TIA. To test our second hypothesis and explore how patient-centered outcomes vary across the spectrum of baseline risk, we build a *treatment outcome model* that determined how treatment influences outcomes after accounting for baseline risk.

### Baseline risk model

Conceptually, the goal of this model was to estimate which factors measured at the time of randomization, influence longer term stroke risk to distinguish “low risk” from “high risk” patients independently of their treatment assignment. Baseline risk models were derived on the whole study population, including both treatment arms as this approach is suggested to improve statistical power without introducing bias [11]. The primary outcome for the *baseline risk model* was selected to best stratify which patients with carotid stenosis were at highest risk from stroke related to that carotid. Thus, our primary outcome was non-perioperative ipsilateral stroke or ipsilateral transient ischemic attack. The decision to combine stroke and TIA was made because some events classified as TIAs in ACAS would likely be classified as strokes today —temporally prolonged TIAs typically have evidence of infarction on MRI imaging and such lesions are now classified as strokes while ACAS was largely conducted in the pre-MRI era [12]. Perioperative events were excluded from the primary model so that the baseline risk construct would not reflect treatment assignment. Instead, these events are accounted for in the *treatment risk model*. Death was omitted from the *baseline risk model* because it was a relatively uncommon outcome and when it occurs is most commonly unrelated to carotid disease. Given that many different notions of “baseline” and to account for changes in event definitions over time risk may be relevant in asymptomatic carotid disease, we also explored alternate baseline risk models for outcomes including: 1. Non-perioperative ipsilateral stroke 2. Any non peri-operative stroke or TIA 3. Any non peri-operative stroke, excluding TIAs.

Factors for consideration in the baseline risk models were identified via a manual literature search to identify factors associated with stroke after CEA. This search identified 15 factors: age [2, 13], sex [14], degree of carotid ipsilateral stenosis, [3, 13] hypertension, [15] hyperlipidemia, [15] diabetes, [16] history of stroke/TIA, [16] coronary artery disease, [6] peripheral vascular disease, [6] chronic renal insufficiency, [17] congestive heart failure [18], bilateral carotid disease, [3] contralateral carotid occlusion [6], smoking [15]. and body mass index [19]. These factors were then mapped to the ACAS dataset. Other factors that putatively predict stroke risk were omitted because they could not be mapped to ACAS, for example, progression of carotid stenosis [20]. In most cases, this mapping was straight-forward (e.g. age), however, in some instances the process required modest additional assumptions. This was most notable for degree of carotid stenosis. In ACAS, patients could be enrolled by varying stenosis criteria, with most qualifying based on Doppler ultrasongraphy and a smaller subset receiving conventional angiography. Patients were assigned to stenosis categories (0–60%, 60–80, 80–100%) for each carotid artery preferentially using the conventional angiography degree (more similar to the NASCET approach than the ECST approach) of stenosis and then the doppler if no angiogram was performed.

For each outcome, backwards selection was used to identify factors for inclusion in the final baseline risk model. Given the number of outcomes in the *primary baseline risk model* (*n* = 122) we were only modestly at risk for over-fitting by including 14 predictor variables in the model prior to selection [21]. Factors were retained in the final model if the *p*-value for a given factor was less than 0.15. To assess and account for over-fitting that can be induced by the variable selection process, models were internally validated by repeating the model with 10,000 bootstrap samples and shrinkage factors were calculated [22–24]. To ensure that the factors selected into the primary model was stable, forward selection models were built by serially including the factors with the strongest univariate associations with each outcome and retained in the final forward-selection model if the addition of those factors reduced the Akaikie Information Criterion (AIC). All models were Cox models and individuals were censored if they died or had a perioperative stroke in model that did not include these elements as part of its outcome definition. Baseline risk was assessed for each patient by estimating the cumulative hazard at median study follow-up duration for each individual.

### Treatment outcomes model

In a second step, we incorporated the baseline risk information into a second model to determine treatment effectiveness on patient-centered outcomes across the spectrum of baseline risk. First, we explored whether relative heterogeneity of treatment effect may exist for any factor included in the *baseline risk model* by determining whether there was an interaction with each factor included in the final risk model and a treatment indicator variable (medical vs. surgical). When no significant interactions were identified, we built a *treatment outcomes model* with a primary outcome of any stroke, TIA or death, including peri-operative events. This outcome was selected because it was considered to be of the overall greatest clinical relevance to patients and consequently if these models were to be used for clinical decision-making, this is the model that would be most relevant to patients. Then we built a Cox model to predict any stroke, TIA or death with dependent variables including baseline risk (determined from the baseline risk model), a treatment indicator and the interaction between baseline risk and treatment. Model discrimination was calculated with Harrell’s c-statistic. Calibration plots were derived for both models by comparing actual and predicted risk, at mean trial follow-up (3.8 years) across quintiles of predicted risk. Average marginal effects were used to estimate risks for treated and untreated patients across the spectrum of baseline risk.

### External validation of baseline risk model

We used data from the Atherosclerosis Risk in Communities (ARIC) cohort component to determine how well the baseline risk model predicted risk in a real world population. The goal of this external validation was to determine wheher our trial-derived ACAS model would have sufficient correlation with real-world risk to allow such a model to be applied in real world practice. In brief, ARIC followed a cohort of individuals aged 45–64 at initial examination in four communities with comprehensive clinical examinations including 4-waves of carotid ultrasound examination between 1987 and 1999, roughly paralleling when ACAS was performed [25]. We identified patients with > 60% carotid stenosis using B-mode ultrasound and applying a previously validated technique [26]. Stroke was identified in ARIC by annually contacting patients, collecting information on hospitalizations and ultimately by nurse abstraction of hospitalization records as previously described. Based on these data, stroke events were classified based on the underlying data as definite/probable embolic or thrombotic ischemic stroke [27]. We then estimated baseline risk in the ARIC population using all baseline risk models derived in ACAS and estimated model discrimination using Harrell’s c-statistic. Calibration was assessed by comparing the predicted risk of stroke (using the secondary baseline risk model with all strokes/no TIAs as its outcome, as this model most closely mapped to the stroke outcome measured in ARIC) with baseline survival from mean trial follow-up in ACAS vs. actual event rates in ARIC.

### Research ethics

The University of Michigan Institutional Review Board reviewed the study protocol and declared it exempt as it relied on de-identified data.

## Results

### Summary of baseline risk models

In the primary *baseline risk model*, the same 3 factors were retained in the forward and reverse selection models: creatinine, body mass index, and degree of contralateral stenosis. All other factors had multivariable *p*-values > 0.15 and failed to reduce the AIC when added to this baseline model.

When secondary outcomes were considered, contralateral stenosis was the only factor that consistently retained across models with a trend towards increased risk for 60–80% stenosis and decreased risk with 80–100% stenosis. For models including all strokes, as opposed to only ipsilateral strokes, other conventional vascular risk factors, such as age, blood pressure, smoking and diabetes, were included (Table 1).

**Table 1:** Summary of Baseline Risk Models. Regression coefficients for continuous variables represent the hazard for a one unit change in each independent variable with the following units: creatinine (mg/dl), bmi (kg/m^2), age (years), DBP (mm Hg)

	Baseline Model — Ipsilateral Stroke/TIA		Secondary Outcome - ipsilateral stroke		Secondary Outcome — All Stroke/TIA		Secondary Outcome - All Stroke	
	Hazard Ratio	*p*-value	Hazard Ratio	*p*-value	Hazard Ratio	*p*-value	Hazard Ratio	*p*-value
Risk Factor								
Contralateral Stenosis 60-80% (ref 0-60%)	1.43	0.13	1.57	0.18	1.37	0.07	1.49	0.09
Contralateral Stenosis 80-100% (ref 0-60%)	0.65	0.12	0.84	0.42	0.81	0.26	0.77	0.34
Creatinine	1.46	0.01			1.31	0.05		
BMI	1.05	0.02	1.05	0.05				
Abnormal Heart Rhythm			0.4	0.12				
Any prior vascular disease (CVD, PVD)			0.59	0.1	1.28	0.08		
Diabetes					1.3	0.1		
Age					1.01	0.27	1.02	0.19
Smoking					1.6	0.002	1.92	0.001
Mean Diastolic BP					1.01	0.05	1.02	0.04
Model Parameters								
N	1551		1575		1622		1648	
Number Outcomes	115		56		215		113	
c-statistic	0.59 [0.54-0.65]		0.63 [0.57-0.71]		0.60 [0.56-0.64]		0.62 [0.56-0.67]	
Model LR test	<0.01		0.03		<0.01		<0.01	
Shrinkage Coefficient	0.85							
External validation c-statistic (ARIC)	0.58 [0.49-0.67]		0.57 [0.47-0.68]		0.49 [0.40-0.59]		0.58 [0.48-0.68]	

### Performance of baseline risk model

**Fig. 1:**
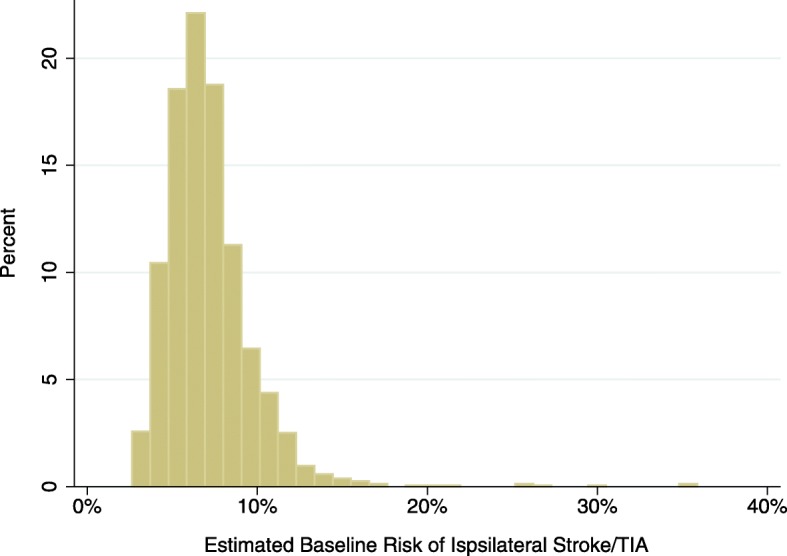
Histogram displaying basleine risk distribution of the primary *baseline risk model* — Estimated Risk of Ipsilateral Stroke/TIA — in the ACAS population

**Fig. 2:**
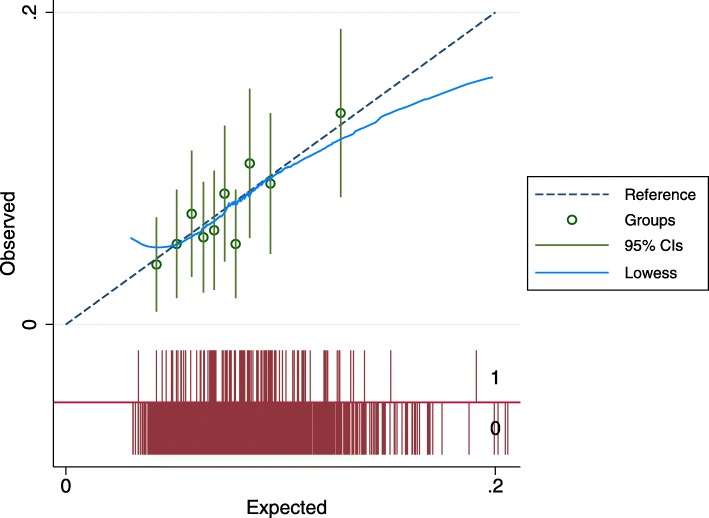
Calibration of the *baseline risk model* in the ACAS population across quintiles of baseline risk

The overall predictiveness of the baseline model was modest with a C-statistic of 0.59 [95% CI 0.54–0.65]. This model also had some evidence of over-fitting as the shrinkage coefficient was 0.85. Individual-level estimated risks at the mean time to last follow-up, from the model, are illustrated in Fig. 1. The lowest risk patients had a model-estimated risk over the course of the trial just under 3% and the highest risk patients estimated risks around 35%. The proportion of the lowest risk quintile with non-perioperative ipsilateral stroke/TIA was 5.1% vs. 12.5% in the highest risk quintile. The primary baseline risk model was well-calibrated compared to actual risk in the lowest two quintile of risk, over-predicted risk in the 3rd quintile and under-predicted in the two highest risk quintiles (Fig. 2**).**

**Fig. 3:**
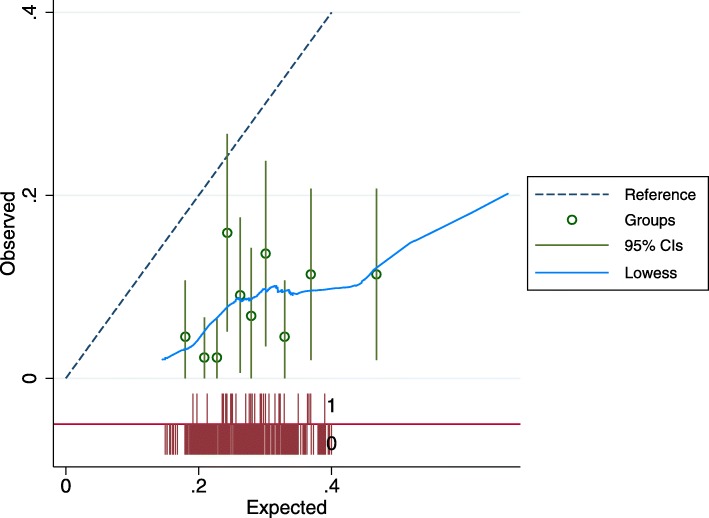
Calibration of the *baseline risk model* (derived in the ACAS population) in the ARIC population

In external validation, the primary baseline risk model had similar predictiveness in ARIC as in ACAS (C-statistic 0.58 vs. 0.58). Models predicting other outcomes had variable performance. The ipsilateral stroke model performed slightly worse in ARIC (0.63 vs. 0.57), the all stroke/TIA model very poorly (0.60 vs. 0.48) and the all stroke model performed about the same (0.61 vs. 0.60) in the internal and external models. However, calibration was poor. In ARIC, 16.4% of the population had a stroke at a mean follow-up of almost 11 years compared to 6.7% at 3.8 years in ACAS (9.2% in the medical arm and 4.5% in the surgical arm). When accounting for the differential length of follow-up by extrapolating the ACAS baseline survival function into the ARIC population, the ACAS model substantially overestimated risk, across the risk spectrum, but particularly in the highest risk patients — 9.1% estimated risk vs. 4.5% actual risk in the lowest risk quintile to 24.6% vs. 10.5% in the highest risk quintile Fig. 3**.**

### Treatment outcome model

**Fig. 4:**
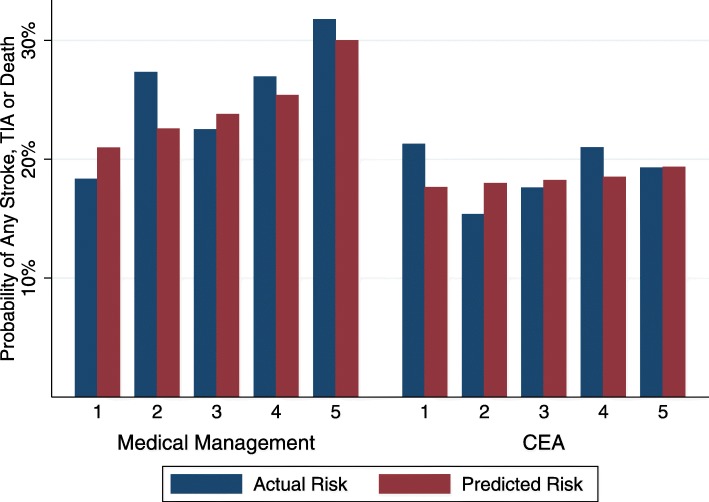
Calibration of the *treatment outcome model* in the ACAS population

**Fig. 5:**
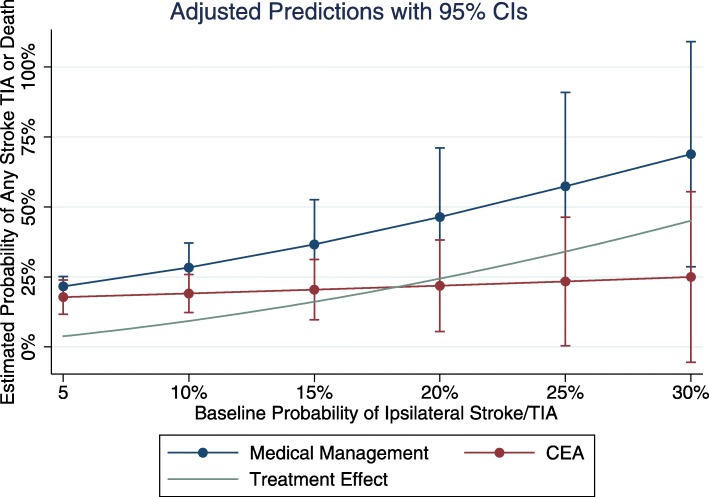
Predicted risk with and without treatment, across the spectrum of basline risk, in ACAS

The *treatment outcome model* was only modestly predictive, C-statistic: 0.56 [0.52–0.60] Predicted risk was reasonably well calibrated to actual risk across the risk spectrum for both CEA and medical management (Fig. 4**)** The predicted risk of any stroke, TIA or death was lower across the baseline risk spectrum with CEA compared to medical management. The interaction term in the model was non-significant (*p* = 0.17), but the direction of the interaction resulted in greater expected relative treatment benefit amongst the pateitns at highest baseline risk. The absolute treatment effect increased across the risk spectrum (Fig. 5) such that the absolute risk reduction in stroke/TIA or death in the lowest risk quintile was 3.2% with CEA vs. 10.7% in the highest risk quintile.

## Discussion

Our risk model using 3 simple clinical factors demonstrates the potential of risk stratification to improve asymptomatic carotid treatment decisions. When applied to the ACAS trial, this model was able to demonstrate that the magnitude of treatment benefit varies 3-fold between the quintile with the greatest and least benefit of CEA. While the ACAS trial was performed some time ago, given the absence of more recent controlled trials in asymptomatic stenosis, they are the best available data to study this question. However, while this model is promising and provides proof of principle, it should not yet be applied to clinical care. Calibration needs to be improved. Our ACAS model overestimated risk in a real-world external validation dataset, ARIC, and the magnitude of overestimation increased in the highest risk subpopulation.

Applying risk prediction models at the bedside requires that models are both sufficiently discriminant and are adequately calibrated. While our baseline risk model reliably identified which patients were at the highest risk (comparable predictiveness), it substantially overestimated the actual level of risk in ARIC (poor calibration). Evaluation of clinical risk models often focuses on predictiveness — how well the model determines which patients are high vs. low risk, often measured with a summary statistic such as the C-statistic. While our model’s discrimination is only moderate, it echoes prior literature that argues that even a moderately predictive model can be clinically actionable [28]. Patients in our model’s top risk quintile have risk more than double the median risk — a group that, if ACAS event rates represented real world event rates, would almost certainly benefit from CEA for asymptomatic disease and likely represents a substantial number of real world patients. However, to apply our model in the real world it must also be adequately calibrated. That is not the case for our model — which provides a striking example of the limitations of poorly calibrated risk models. The average patient in ACAS has about a 7% baseline risk of stroke which translates into an approximately 5% absolute risk reduction in stroke or death over a median of 3.7 years of follow-up with CEA over medical management. If, however, an apparently similar patient outside the trial had a true baseline risk that was half as high as suggested by our ARIC external validation (i.e. 3.5%), that would translate into an absolute risk reduction around 1% over a similar time frame. Given that many have argued that asymptomatic CEA is ill-advised even if the ACAS effect size was realistic, a 5-fold smaller effect would substantially alter the treatment calculus. The problem with clinically applying a poorly calibrated model is that its difficult to know which calibration standard to apply to a given population. If the ARIC standard is more realistic, then asymptomatic CEA should likely be used sparingly, if at all. If the ACAS standard is more realistic, then asymptomatic CEA likely represents a reasonably effective treatment option for a substantial fraction of patients with asymptomatic carotid stenosis.

Both medical [29] and surgical [30] treatment have improved considerably since ACAS, and ongoing trials will clarify several key parameters relevant to decisions regarding asymptomatic CEA (i.e. expected event rates accounting for improvements in best medical and surgical treatments.) [31] Yet, if outcome-driving factors influence trial seletion, as may be the case with ACAS and ARIC, ongoing trials may not inform the anticipated value of treatment in the unselected population. Assessing external validity is a challenge for all trials. But, it is uniquely challenging for asymptomatic carotid disease because patients are not selected via population-based screening (as in ARIC), but variably in the context of either non-vascular symptom presentations (e.g. syncope) or via screening in the context of comorbid vascular disease. Clinical registries may mitigate some such selection biases by reflecting treatment decisions in the real world as opposed to the trial enviornment, however, they still can not account for the possibility that real world patients are likely much higher risk that patients that would have been identified by population-based screening. Even if modern trials provide clear and convincing evidence of the benefits of treatment in modern asymptomatic populations, the question of which population the results apply to will remain a challenge.

The problem of adequately discriminant, but poorly calibrated, risk models is common. For example, the widely used atherosclerotic cardiovascular disease (ASCVD) risk score has varying calibration depending on the study population [32]. Yet, poor calibration does not necessarily preclude application of such models. Instead, it may simply require that the model be recalibrated to the target population. In the case of ASCVD, prior work has shown that simple recalibration techniques (e.g. estimating new model intercepts or re-estimating model coefficients) represent a plausible strategy to maintain the conceptual framework of the underlying model while generating well-calibrated predictions. For example, Sussman et al. have found that while the ASCVD risk models substantially over-estimated risk (resulting in unduly aggressive treatment recommendations) in a Veteran Affairs population, but that simple recalibration that maintained the conceptual framework of ASCVD (and even the model coefficients) resulted in vastly improved calibration and more accurate treatment decisions [33]. Applying a similar approach to our carotid models is generally feasible in an era of Big Data and has the potential to substantially inform carotid decision-making by applying 3 steps 1. For a given target population (e.g. clinic, health system, hospital), extract real world data on outcomes in patients with asymptomatic carotid stenosis, likely using electronic medical record data. 2. Fit a model by including the predictor variables in our model independently (completely refitting a model in the target population) or by calculating the linear predictor of our model (fitting a new intercept in the target population) and do the same for perioperative risk using previously developed models. 3. Verify model predictiveness and calibration in the target population. While the poor calibration of our model precludes direct clinical application there is considerable potential to apply this technique to improve carotid decisions after appropriate recalibration.

Understanding the poor calibration of our model may inform carotid decision-making. A number of factors may contribute to the poor calibration of the ACAS model in ARIC. First, measurable patient characteristics differ between samples —the mean age in ACAS (67) is considerably greater than the mean in our ARIC sample (62). However, age does not strongly predict stroke risk in either sample. A more likely explanation is that ACAS patients were at higher baseline risk due to clinical factors that led to them undergoing carotid screening in the first place. In ARIC, carotid stenosis was detected by screening comprehensively a large population regardless of baseline risk. In ACAS, conversely, patients received carotid testing based on real-world clinician practice, probably due to patients having some underlying clinical condition, thereby selecting fo a population at considerably higher risk of stroke. Another possibility is that risk factor management differed between studies, but this seems relatively unlikely given that both studies were conducted in similar epochs, and, if anything, we would have anticipated that trial participants would have more intense risk factor management than patients in the community. Yet another possibility, is that the stroke-definition or definition of carotid stenosis was more sensitive in ACAS than in ARIC. More broadly, the differences in stroke risk between screening-identified cohort and a clinically-identified cohort illustrates one of the key challenges in treating patients with asymptomatic stenosis. It is likely that the rationale for detection influences stroke risk, but the mechanisms underlying that increased risk are not well known.

While poor calibration is the primary barrier to the clinical application of these models, predictiveness is also only modest and is considerably worse than in prior work [34]. This likely reflects several factors. First, the clinical factors surrounding the symptomatic event (e.g. amaurosis vs. TIA vs. stroke and time from event) are key predictors in the symptomatic context, but are not relevant to to asymptomatic patients. Second, plaque charateristics (e.g. ulcerated plaque) were also important predictors in the symptomatic models, but were not available in most ACAS patients because they qualified for the trial on the basis of ultrasound evaluations that are insensitive to these features compared to the more sensitive angiograms that were applied in the symptomatic trials. It is plausible that other plaque factors are also important predictors (e.g. plaque length, location, deree of calicifcation) but can not be included in this model because they were not systematically measured in the non-surgical arm in ACAS. Finally, ipsilateral degree of stenosis is a strong predictor in the symptomatic context, but not the asymptomatic context. The reasons why this is the case are unclear and numerous possible explanations exist (e.g. measurement error and limited statistical power). It is also possible that the “true” story about the relationship between degree of stenosis is complex. For example, plaque characteristics and degree of stenosis may interact to increase risk. Amongst symptomatic patients, then, the fact that they have had an event results in selection of group with a combination of high risk plaque features such that ipsilateral stenosis is an important predictor whereas amongst asymptomatic patients have, on the whole, lower risk plaque characteristics such that ipsilateral stenosis is a weak predictor. An additional limitation of our models is that the factors selected for the models were not the factors with our strongest prior probabilities (e.g. contralateral stenosis selected into the model instead of ipsilateral stenosis). Similarly, the coefficients associated with these factors does not fit strongly with our priors (i.e. higher risk with 60–80% contralateral stenosis vs. 80–100% contralateral stenosis). These findings suggest that the credibility of our models should be judged carefully and that they may reflect some combination of random error, overfitting, omitted variable bias or confounding.

## Conclusions

Our risk model using 3 simple clinical factors demonstrates the potential of risk stratification to improve asymptomatic carotid treatment decisions. However, poor calibration on external validation limits its immediate and direct clinical applicability.

## Data Availability

The ACAS dataset is available, upon request and application, from NINDS: https://www.ninds.nih.gov/Current-Research/Research-Funded-NINDS/Clinical-Research/Archived-Clinical-Research-Datasets. ARIC is available after application from: https://sites.cscc.unc.edu/aric/desc_pub.

## Abbreviations

ACAS: Asymptomatic Carotid Atherosclerosis Study
AIC: Akaikie Information Criterion
ARIC: Atherosclerosis Risk in Communities
ASCVD: Atherosclerotic cardiovascular disease
BMI: Body Mass Index
CEA: Carotid endarterectomy
NNT: Number needed to treat
TIA: Transient ischemic attack
